# Screening of Durum Wheat (*Triticum turgidum* L. subsp. *durum* (Desf.) Husn.) Italian Cultivars for Susceptibility to Fusarium Head Blight Incited by *Fusarium graminearum*

**DOI:** 10.3390/plants10010068

**Published:** 2020-12-31

**Authors:** Gaetano Bentivenga, Alfio Spina, Karim Ammar, Maria Allegra, Santa Olga Cacciola

**Affiliations:** 1 Valagro S.p.A., via Cagliari, 1, 66041 Atessa, Chieti, Italy; gaetano.bentivenga@virgilio.it; 2 Agricultural Research Council and Economics (CREA)–Research Centre for Cereal and Industrial Crops, Corso Savoia, 190, 95024 Acireale, Italy; alfio.spina@crea.gov.it; 3 International Maize and Wheat Improvement Center (CIMMYT), Km. 45, Carretera México-Veracruz, El Batán, Texcoco 56237, Mexico; k.ammar@cgiar.org; 4 Agricultural Research Council and Economics (CREA)–Research Centre for Olive, Fruit and Citrus Crops, Corso Savoia 190, 95123 Catania, Italy; maria.allegra@crea.gov.it; 5 Department Agriculture, Food and Environment (Di3A), University of Catania, via S. Sofia n.100, 95123 Catania, Italy

**Keywords:** phenotypic traits, FHB index, mycotoxins, flowering biology, plant height, qualitative features

## Abstract

In 2009, a set of 35 cultivars of durum wheat (*Triticum turgidum* L. subsp. *durum* (Desf.) Husn.) of Italian origin was screened for fusarium head blight (FHB) susceptibility at CIMMYT (Mexico) and in the 2019–20 cropping season, 16 of these cultivars, which had been included in the Italian National Plant Variety Register, were tested again in southern and northern Italy. Wheat cultivars were artificially inoculated during anthesis with a conidial suspension of *Fusarium graminearum sensu lato* using a standard spray inoculation method. Inoculum was a mixture of mono-conidial isolates sourced in the same areas where the trials were performed. Isolates had been characterized on the basis of morphological characteristics and by DNA PCR amplification using a specific primer set and then selected for their virulence and ability to produce mycotoxins. The susceptibility to FHB was rated on the basis of the disease severity, disease incidence and FHB index. Almost all of the tested cultivars were susceptible or very susceptible to FHB with the only exception of “Duprì”, “Tiziana” and “Dylan” which proved to be moderately susceptible. The susceptibility to FHB was inversely correlated with the plant height and flowering biology, the tall and the late heading cultivars being less susceptible.

## 1. Introduction

Fusarium head (ear) blight (FHB) or scab, a fungal disease affecting kernel development, is one of the most damaging diseases of cereal crops such as wheat, barley and oats worldwide. It affects both durum wheat [*Triticum turgidum* L. subsp. *durum* (Desf.) Husn.] and bread wheat (*Triticum aestivum* L. subsp. *aestivum*) and poses a challenge for wheat breeders worldwide [1]. Durum wheat is the most widespread staple crop in the Mediterranean area and the 10th most important crop in the world, with about 8% coverage of the wheat area [2,3]. It represents a strategic commodity for several producing areas over the world, including the Mediterranean basin, North America’s Great Plains, the desert area of South-Western United States, Northern Mexico’s Yaqui Valley (state of Sonora) and India [4]. The disease is caused by several species of *Fusarium*, whose infections result in similar symptoms. The *Fusarium graminearum* Schwabe (telomorph: *Gibberella zeae* Schw. (Petch)) species complex is the prevalent causal agent of FHB worldwide and in hotter climatic areas [5,6,7]. The first symptoms of FHB occur shortly after flowering and consist in premature bleaching of spikelets, which in a more advanced stage of the disease may extend to the entire head causing serious yield losses. Moreover, FHB infections reduce the grain quality due to the production of toxins by the pathogen, mainly deoxynivalenol (DON), nivalenol (NIV) and their acetylated derivatives, which contaminate foods and feeds and are harmful for humans and domestic animals [8,9,10]. There can be chronic toxicity due to consumption of these mycotoxins in contaminated food. Type-B trichothecenes, such as DON, are phytotoxic and can act as virulence factors on wheat. As a matter of fact, a positive correlation was often observed between the production of these mycotoxins and FHB severity [11]. The disease is particularly severe in humid and moderately warm areas and causes severe epidemics on bread wheat worldwide [12]. 

Zero and minimum tillage increase FHB severity in areas where wheat is grown after maize, an alternate host for the fungus. Infected crop residues are the primary source of inoculum, such as ascospores and conidia. Italy ranks first for production of durum wheat in Europe and second in the world. The national production accounts for about 4.4 million tons in 2015 and FHB causes serious problems in northern and central regions [13].

The most cost-effective and environmentally safe method to manage the disease would be the use of resistant and/or tolerant wheat genotypes obtained by conventional and innovative breeding strategies. Unfortunately, breeding programs are hindered by the fact that resistance to FHB is under polygenic inheritance. Furthermore, tests in different environmental conditions are needed to verify the susceptibility of wheat germplasm to FHB as the severity of the disease is influenced by climatic conditions and may result in a large variability of the interaction between genotype and environment [5,14]. Over 500 quantitative trait loci (QTLs) conferring small to moderate effects on the different FHB resistance types have been reported in wheat [15,16]. 

Sources of resistance were identified in bread wheat genotypes, like the Chinese cultivar “Sumai #3”, the Brazilian genotype “Frontana” and the line “Prag 8” from Eastern Europe [17]. Other sources of resistance were found in species of the *Triticeae* tribe, like *Elymus giganteus* L. (syn. *Leymus racemosus* Lam., 2n = 4x = 28 JJNN), *Roegneria kamoji* C. Koch (syn. *Agropyron tsukushiense* Honda, 2n = 6x = 42StsStsHtsYts) and *Roegneria ciliaris* (Trin) Nevski (syn. *Agropyron ciliare* (Trin.) Franchet, 2n = 4x = 28, ScScYcYc) [18,19,20,21,22]. The last two species originated in southern China, a region characterized by a wet and warm climate [23]. Introgression of resistance genes from *Thinopyrum junceiforme* into durum wheat through hybridization was also pursued [24]. So far, however, very few sources of resistance have been identified in durum wheat [23]. Up to six types of resistance to FHB have been described [25]: resistance to initial infection (Type I); resistance to the spread of the infection within the spike (Type II); ability of the host to degrade (Type III) and tolerate (Type IV) DON; resistance to kernel infection (Type V); tolerance to FHB (Type VI).

Identification of molecular markers associated with QTLs for FHB resistance makes it possible marker assisted selection (MAS), which could be a useful tool for breeders. So far, several studies concerning QTLs maps have been performed, mainly using sources of resistance originating from Asia, like the cultivars “Sumai #3”, “Wangshuibai” and “Wuhan-1” and one of the main QTLs is “Qfhs.ndsu-3BS”, located on the short arm of chromosome 3B [26,27,28,29,30,31,32]. To date, however, sources of resistance conferring complete resistance to FHB have not been identified and many of the original sources of resistance are not well adapted to all wheat production areas of the world. Some success has been achieved in transferring FHB resistance from exotic sources into cultivars with desired traits. 

Another strategy, which could be crucial to both conventional and modern molecular breeding approaches, is the identification and exploitation of FHB resistance already present in local germplasm. In this respect, the Italian germplasm of durum wheat, which has been traditionally selected for other traits such as yield potential, grain quality, drought and heat tolerance, plant height, earliness and resistance to rusts, has been episodically and only very preliminarily evaluated for FHB resistance [33,34,35,36,37,38,39,40,41,42,43,44]. 

The objective of this study was to test the susceptibility of a wide set of Italian durum wheat cultivars to FHB in different environments, under a high inoculum pressure and conditions conducive for the disease, using an internationally recognized standard assay method. 

## 2. Results

Field tests using a standard assay method in three very different and distant geographical areas provided information on the susceptibility to FHB of a large pool of Italian cultivars of durum wheat and showed a significant correlation between the susceptibility to the disease and other phenotypic traits. The disease susceptibility indices as well as the agronomic and grain quality traits of the 35 durum wheat cultivars and six bread wheat accessions used as references tested at CIMMYT in Mexico are shown in Table 1 and Table 2, respectively. Table 1 summarizes the results of pathogenicity tests to evaluate the susceptibility of wheat cultivars to FHB. 

The overall mean proportion of damaged kernels on forty-one cultivars (35 of durum wheat and six of bread wheat) was 16.2%, the highest percentage (32.2%) being found in the durum wheat cultivar “Summa”. “Daunia”, “Grecale”, “Plinio”, “Poggio”, “Saadi” and “Simeto” also showed a high percentage of damaged kernels (>20%). “Duprì”, “Tiziana” and “Dylan”, which were characterized by low values of disease severity, showed proportions of damaged seeds not exceeding 12%. Not surprisingly, the lowest proportions of damaged seeds were found in the reference cultivars of bread wheat “Sumai #3”and “Gondo/CBRD”, which are known to be FHB resistant and “Heilo”, which is known to be moderately tolerant. 

The average disease severity (DS) of all wheat accessions was high (30.3%). The reference cultivars of bread wheat “Sumai #3” and “Gondo/CBRD”, known to be resistant to FHB, showed very low DS values (2.1 and 3.6%, respectively), as expected. Of the Italian durum wheat cultivars “Duprí”, “Tiziana” and “Dylan” showed the lowest DS values (9.4, 13.5 and 15.6%, respectively). The highest DS value was found, as expected, for the reference cultivar of bread wheat “Gamenya” (54.5%), which is known to be very susceptible to FHB. High DS values were also shown by the durum wheat cultivars “Arcangelo”, “Creso”, “Daunia”, “Duilio”, “Iride”, “Italo”, “Meridiano”, “Nibbio”, “Ramsete”, “Saadi”, “Ulisse”, “Vettore” with values ranging from 34.5 to 42.5 and, above all, “Plinio” and “Summa” (46.9 and 46.8%, respectively). All are early to medium–early cycle cultivars (Table 2). The mean DS values of other durum wheat cultivars were close to the overall mean value.

The average disease incidence (DI) in all wheat accessions was very high (92.32%), indicating the inoculation method used in the susceptibility assay was effective. The reference cultivars of bread wheat “Sumai #3” and “Gondo/CBRD” showed the lowest DI values (35.0 and 55.0%, respectively), as expected, while out of durum wheat cultivars the lowest DI values were observed for “Duprí”, “Dylan” and “Tiziana” (65.0, 67.5 and 67.5%, respectively). The DI values in the cultivars “Nerone”, “Levante”, “Durango”, “Piceno” and “Virgilio” ranged from 87.5% to 92.5%. The remaining durum wheat cultivars showed disease incidence values ranging between 95.0 and 100.0%. 

The morpho-physiological, agronomic and grain qualitative features of the 35 durum wheat cultivar and six bread wheat accessions tested at CIMMYT in Mexico, are shown in Table 2.

Values of FHB index revealed significant variability. Only the Italian durum wheat “Duprì”, “Tiziana” and “Dylan”, with a rather long cycle (88–90 days from emergence to heading) showed low FHB indices (6.2, 9.0 and 10.6, respectively). The FHB index values of 10 cultivars, including “Bravo”, “Campodoro”, “Crispiero”, “Gabbiano”, “Levante”, “Nerone”, “Piceno”, “Romano”, “Saragolla” and “Virgilio” ranged from 19.4 of “Romano” to 27.2 of “Crispiero”. Accessions with high FHB index values were the majority and included “Arcangelo”, “Creso”, “Daunia”, “Duilio”, “Durango”,”Fortore”, “Grecale”, “Iride”, “Italo”, “Meridiano”, “Nibbio”, “Peleo”, “Perseo”, “Plinio”, “Poggio”, “Ramsete”, “Saadi”, “Simeto”, “Summa”, “Tresor”, “Ulisse” and “Vettore”. The most susceptible were “Plinio” and “Summa”, with FHB index values of 46.9 and 46.8, respectively. As far as the six reference genotypes of bread wheat were concerned, “Gamenya” was the most susceptible (FHB index value 54.5), “Falcin”, “Heilo” and “Ocoroni F86” showed relatively high FHB index values (23.7, 27.3 and 31.4, respectively), while “Sumai #3” and “Gondo/CBRD” proved to be much less susceptible (FHB index values 0.65 and 1.95, respectively), according to the susceptibility scale already known for these cultivars used as a reference. 

Table 2 summarizes the main phenological and grain qualitative features of the wheat cultivars tested, including heading time (HT), plant height (PH), physiological maturity (PM), seeds per spike (SS) and thousand kernels weight (TKW). The HT ranged from 11 days, for “Daunia”, “Ramsete” and “Summa” and also for the reference cultivars “Falcin” and “Ocoroni F86” (earliest), to 31 days for “Bravo” and “Durango” (very late). The average HT of all wheat genotypes was 19.2. The HT of “Duprì”, “Tiziana” and “Dylan” (medium-late flowering cultivars) in the experimental conditions at CIMMYT were in average 26, 22 and 22 days, respectively. 

The average PH of all durum wheat cultivars tested was 67.1 cm. The height of most cultivars did not differ consistently from the average height of all cultivars tested. Only two cultivars, “Durango” and “Nerone”, stood out above all others for their height (80 cm). Fourteen cultivars, including “Arcangelo”, “Creso”, “Daunia”, “Dylan”, “Iride”, “Italo”, “Peleo”, “Perseo”, “Plinio”, “Poggio”, “Ramsete”, “Simeto”, “Vettore” and, above all, “Tresor” were smaller in size (mean PH < 65 cm). Of the reference varieties of bread wheat, “Sumai #3”and “Gondo/CBRD” were the tallest in size (PH 105.0 and 91.5 cm, respectively). The other four tested varieties did not differ consistently from the average height of all durum wheat cultivars tested. PM was reached early in almost all cultivars. The mean value of the cultivars with leaf area index (LAI) equal to zero was 66.1 days after August 1st. Seven durum wheat cultivars, “Duprì”, “Durango”, “Levante”, “Nerone”, “Romano”, “Saragolla” and “Simeto” and the reference cultivar of bread wheat “Sumai #3”, reached PM very late (74 days after August 1st).

A synoptic table of factorial ANOVA across the means of the disease susceptibility indices and some morpho-physiological traits correlated to FHB susceptibility of the 16 Italian durum wheat cultivars and two bread wheat reference cultivars tested in two different sites in northern and southern Italy, is reported in Table 3, while a synoptic table of factorial ANOVA across the means of the agronomic and qualitative traits is reported in Table 4.

The two sites differed significantly for almost all the variables at *p* ≤ 0.001, with the exception of disease incidence and hectoliter weight, which were significant at *p* ≤ 0.01. The values of almost all variables were always higher at Eraclea (northern Italy) than at Reggio Calabria (southern Italy) except for the hectoliter weight and the protein content. With regards to the variability of factor B (cultivar), all the differences between the variables were highly significant (*p* ≤ 0.001). Similarly, with regards to the variability factor A x B (site x cultivar) interaction, all the variables were highly significant (*p* ≤ 0.001). In the trial performed at Eraclea (northern Italy), the overall mean proportion of damaged kernels for the 18 cultivars tested was 18.3%. As expected, the highest (27.2%) and the lowest (5.1%) proportions of damaged kernels were found in the bread wheat cultivars “Gamenya” and “Sumai #3”, respectively, both included in the trial as references. As for the durum wheat cultivars, the highest proportions of damaged kernels were found in the varieties “Simeto”, “Grecale”, “Saadi”, “Italo”, “Duilio and “Vettore”, with values >20%. Conversely, “Tiziana”, “Dylan”, “Creso” and “Duprì” were characterized by low proportions of damaged seeds not exceeding 16%. The overall mean disease severity of all wheat varieties was 32.8%. Not surprisingly, the lowest and the highest disease severity values (2.6 and 55.1%) were found in the reference bread wheat cultivars “Sumai #3” and “Gamenya”, respectively. Even in this trial, like in Mexico, the durum wheat cultivars “Duprí”, “Tiziana” and “Dylan” showed relatively low disease severity values (12.4, 13.6 and 14.4%, respectively). Conversely, “Saadi”, “Iride” “Meridiano”, “Italo”, “Vettore” and “Tresor” showed high disease severity values, ranging from 39.7%, for “Tresor”, to 44.8% for “Saadi”. The overall mean disease incidence of the 18 wheat accessions was quite high (91.9%). The reference cultivar of bread wheat “Sumai #3” showed the lowest disease incidence value (50.0%) while the value for the highly susceptible reference cultivar “Gamenya” was 100%. Of the durum wheat cultivars “Tiziana”, “Duprí and “Dylan” showed the lowest disease incidence values (67.7 and 71.0%, respectively) while the disease incidence was 95.0% for “Virgilio”. In all the other durum wheat cultivars the disease incidence was 100%. Like in Mexico, even in this trial the values of FHB index among the tested cultivars varied greatly. Not surprisingly, the reference accession of bread wheat “Gamenya” was the most susceptible, with an FHB index value of 55.1%, while “Sumai #3” proved to be the least susceptible, with an FHB index value of 1.3%. Of the durum wheat cultivars, only “Duprì”, “Tiziana” and “Dylan” showed relatively low FHB indices (8.8, 9.2 and 10.3%, respectively). The values of FHB index of “Saragolla” and “Grecale” were intermediate (28.3 and 29.4%, respectively), while all the other durum wheat cultivars tested showed high FHB index values, ranging from 33.4% for “Virgilio” to 44.8% and 44.6% for “Saadi” and “Iride”, respectively.

The HT of all wheat genotypes was in general longer than in Mexico and ranged from 23.7 days for “Italo” and “Tresor” to 37.3 for “Tiziana”. The bread wheat cultivars “Gamenya” and “Sumai #3” were very late (41.0 and 41.7 days, respectively). The overall mean PH was 87.3 cm. The height of the cultivars ranged from 78 cm for “Creso” to 91.7 cm for “Fortore”. The reference cultivars of bread wheat “Sumai #3”and “Gamenya” were the tallest (127.0 and 106.7 cm, respectively). 

In the trial performed at Reggio Calabria the overall mean proportion of damaged kernels for the 18 cultivars tested was 16.8%. Not surprisingly, the highest (24.6%) and the lowest (4.5%) proportions of damaged kernels were shown by the bread wheat cultivars “Gamenya” and “Sumai #3”, respectively, both included in the trial as references. As regards the durum wheat cultivars, the highest proportions of damaged kernels were shown by the cultivars “Saadi”, “Italo”, “Grecale” and “Duilio, with values >20%. Conversely, “Duprì”, “Tiziana” and “Dylan” were characterized by low values of DS, with proportions of damaged seeds not exceeding 15%. The overall mean DS of all wheat accessions was 29.6%. As expected, the lowest and the highest DS values (1.0 and 53.3%, respectively) were shown by the reference cultivars of bread wheat “Sumai #3” and “Gamenya”, respectively. Even in this trial the durum wheat cultivars “Duprí”, “Tiziana” and “Dylan” showed relatively low DS values (10.4, 12.3 and 13.5%, respectively). Conversely, the cultivars “Italo”, “Meridiano”, “Iride”, “Creso”, “Duilio” and “Tresor”, showed relatively high DS values, ranging from 33.7%, for “Tresor”, to 39.9%, for “Italo”. 

The overall mean DI of the 18 wheat accessions was quite high (89.7%). The reference cultivar of bread wheat “Sumai #3” showed the lowest DI value (45%) while the value for the highly susceptible reference cultivar “Gamenya” was 100%. Of the durum wheat cultivars “Duprí”, “Tiziana” and “Dylan” showed the lowest DI values (66.7, 67.0 and 70.0%, respectively) while the DI was 90.0% for “Simeto” and 91.7% for both “Saragolla” and “Virgilio”. In all the other durum wheat cultivars the DI ranged between 96.7 and 100%. 

Even in this trial, the values of FHB index varied greatly. The reference cultivar of bread wheat “Gamenya” was the most susceptible, with an FHB index value of 53.3%, while “Sumai #3” proved to be the least susceptible, with an FHB index value of 0.8%, in accordance with the susceptibility scale already known for these cultivars. Of the durum wheat cultivars, only “Duprì”, “Tiziana” and “Dylan”, showed relatively low FHB indices (6.9, 8.2 and 9.5%, respectively). The values of FHB index of “Saragolla” and “Virgilio” were intermediate (21.3 and 21.9%, respectively), while all the other durum wheat cultivars tested showed high FHB index values, ranging from 27.5% for “Grecale” to 41.5% for “Saadi”. 

The overall mean of HT was 24.6 days. It ranged from 19 days for “Tresor” to 32.3 days for “Tiziana” and the two bread wheat cultivars “Gamenya” and “Sumai #3” that were very late (37.3 and 36.3 days, respectively).

The overall mean of PH was 83.8 cm. The height of most cultivars did not differ consistently from the overall mean. “Duprì”, “Saadi”, “Creso” and “Iride” were relatively small in size (mean PH < 75 cm), while the two cultivars of bread wheat, “Sumai #3” and “Gamenya” were the tallest (123.4 and 102.4 cm, respectively). 

The agronomic and grain qualitative traits of the wheat tested at Eraclea (Veneto region) and Reggio Calabria (Calabria region), included grain yield (GY) and protein content (PC), besides physiological maturity (PM), hectoliter weight (HW) and thousand kernels weight (TKW), are reported in Table 4. 

In Eraclea the overall mean of GY of the 18 tested wheat cultivars was 4.6 t ha^−1^ ranging from 3.5 t ha^−1^ for “Fortore” to 6.0 t ha^−1^ for “Sumai #3”. Of the durum wheat cultivars “Tiziana”, “Virgilio”, “Dylan”, “Iride” and “Meridiano” showed grain yield ≥5.0 t ha^−1^. The mean value of PM was 73.0 days after April 1st. The reference cultivar of bread wheat “Sumai #3” reached PM very late (79.7 days after April 1st). Even five durum wheat cultivars, “Creso”, “Tiziana”, “Vettore”, “Saadi” and “Virgilio” were late (mean PM value 75.8 days after April 1st). The overall mean of HW was 79.3 kg hL^−1^. As regards durum wheat varieties HW ranged from 78.1 for “Meridiano” to 82.4 kg hL^−1^ for “Saadi”, while it was relatively low for the bread wheat cultivars (mean value 73.4 kg hL^−1^). The overall mean of TKW was 40.6 g. As far as the durum wheat varieties were concerned TKW values ranged from 33.2 g for “Saadi” to 48.1 g for “Simeto”, while both bread wheat cultivars “Sumai #3”and “Gamenya” showed lower values (33.2 and 26.5 g, respectively). The overall mean of PC was 12.9%. The durum wheat cultivar “Dylan” and the reference bread wheat cultivar “Sumai #3” showed the lowest levels of proteins (11.4 and 10.8%), which were associated with high grain yields. The protein content of most cultivars did not differ consistently from the overall mean of all cultivars tested, with the only exception of “Duprì” that showed a protein content of 16.1%.

The overall mean of GY at Reggio Calabria was 4.0 t ha^−1^ with a range from 2.7 t ha^−1^ for “Duprì” to 5.3 t ha^−1^ for the reference cultivar “Sumai #3”. Of the durum wheat cultivars “Tiziana”, “Virgilio” and “Meridiano” showed the lowest GY values (4.7, 4.7 and 4.6 t ha^−1^, respectively). The mean value of the cultivars with LAI equal to zero was 66.1 days after April 1st. Only two varieties, “Duilio and “Grecale” were very early (58.3 and 59.3 days from April 1st, respectively). Eight durum wheat cultivars, “Vettore”, “Creso”, “Tiziana”, “Saadi”, “Virgilio”, “Dylan”, “Italo” and “Meridiano” and the reference cultivar of bread wheat “Sumai #3”, reached PM late (mean PM value 65.2 days after April 1st). 

The overall mean of HW was 75.0 kg hL^−1^. In durum wheat cultivars it ranged from 78.0 kg hL^−1^ for “Meridiano” to 82.6 kg hL^−1^ for “Duilio”, while, as in the trial carried out in northern Italy, the reference cultivars of bread wheat showed the lowest values (mean 73.0 kg hL^−1^). The overall mean of TKW was 36.9 g. TKW values of the durum wheat cultivars ranged from 32.2 g for “Saadi” to 47.7 g for “Simeto”, while, once again, the two cultivars of bread wheat showed the lowest values (33.2 g and 26.4 g, respectively). The overall mean of PC was 12.4%. The PC of durum wheat cultivars was medium-to-high for all genotypes. “Duprì” was the cultivar with the highest protein content (16.3%), consistently with the results of the other two trials, while the two bread wheat cultivars “Gamenya” and “Sumai #3” showed the lowest PC (mean value 11.4%). 

In both trials performed in Italy a highly significant positive correlation also was found between the FHB index and the DS, with an r value equal to 0.9891 and 0.9972 (data not shown), at Reggio Calabria and Eraclea, respectively, confirming that these two parameters can be used alternatively with each other to express the susceptibility level of wheat cultivars to FHB.

**Figure 1:**
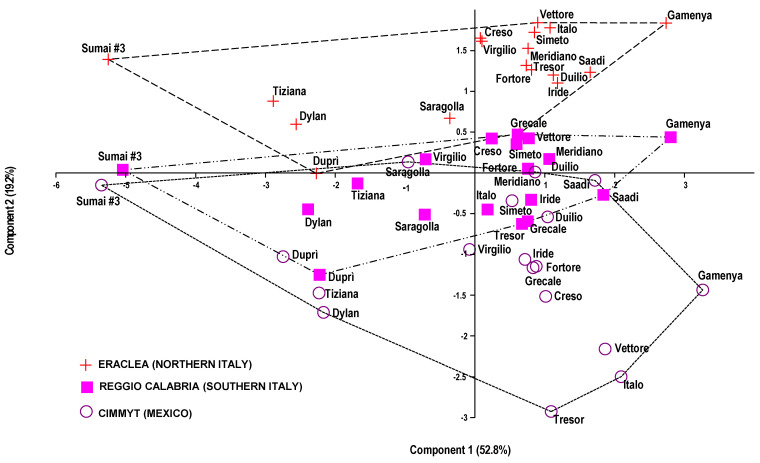
Scatter diagram resulting from principal component analysis (PCA), as defined by the first two principal components. Cultivars are clustered on the basis of similarity of disease susceptibility indices, phenological traits and qualitative grain features. They are grouped by site: Eraclea, Reggio Calabria (RC) and CIMMYT. Groups are delimited by convex hulls to highlight their respective trends.

With the aim of summarizing the results, a Principal Component Analysis (PCA) was carried out on the seven parameters common to all three experimental sites, CIMMYT (Mexico), Eraclea (northern Italy) and Reggio Calabria (southern Italy), i.e., proportion of damaged kernels, disease severity, disease incidence, FHB index, plant height, physiological maturity and thousand kernels weight (Figure 1). 

**Figure 2:**
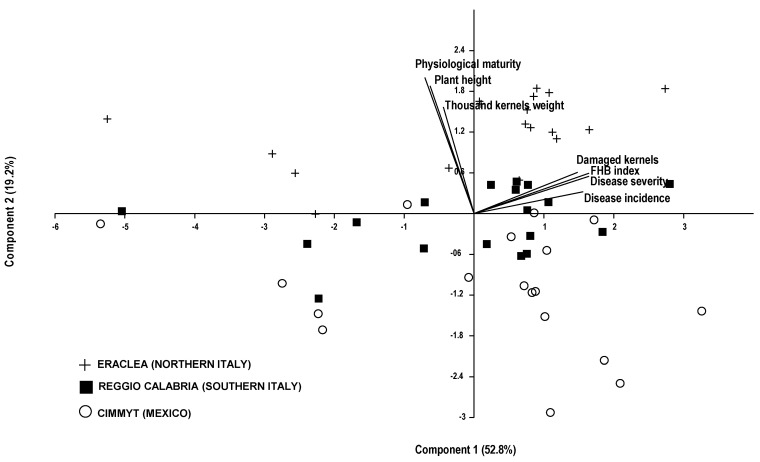
Principal component analysis (PCA) biplot. Cultivar scores are grouped by site (Eraclea, Reggio Calabria and CIMMYT); vectors represent seven variables, including four disease susceptibility indices as well as three morpho-physiological and grain qualitative features.

Two principal components, representing 72.0% of the explained variance, have been extracted. PC1 (52.8% of the total variance) was highly correlated to FHB index, disease severity, disease incidence and proportion of damaged kernels (0.949, 0.948, 0.899 and 0.855, respectively) (Figure 2) resulting in being an indicator of cultivar susceptibility to the disease. 

With respect to PC1, “Sumai #3” and “Gamenya”, the two bread wheat cultivars used as references, exhibited opposite behaviors, consistently with expectations. “Gamenya”, in fact, showed the highest positive scores and “Sumai #3” the lowest ones, confirming the former cultivar is very susceptible to FHB while the latter is tolerant. The durum wheat cultivars that showed the highest scores, i.e., a greater susceptibility to FHB, were “Italo” (2.092), “Vettore” (1.863) and “Saadi” (1.718) at CIMMYT, “Saadi” (1.649), “Iride” (1.183) and “Duilio” (1.122) at Eraclea and “Saadi” (1.839) at Reggio Calabria. Conversely, the lowest scores, were assigned to “Duprì” (−2.748), “Tiziana” (−2.233) and “Dylan” (−2.170) at CIMMYT, again “Tiziana” (−2.890), “Dylan” (−2.562) and “Duprì” (−2.272) at Eraclea, “Dylan” (−2.389) and “Duprì” (−2.225) at Reggio Calabria, indicating a lower susceptibility of these cultivars to the disease. PC1 did not discriminate the experimental site on the basis of the disease susceptibility of the grain. PC2 (19.2% of the total variance) was correlated to physiological maturity, plant height and thousand kernels weight (0.695, 0.652 and 0.545, respectively) and consequently it was an indicator of phenological as well as grain qualitative features of the cultivars. With respect to the PC2 axis, the data sourced from each of the three experimental sites, CIMMYT, Eraclea and Reggio Calabria, clustered on the basis of the expression of phenological and grain qualitative parameters and at Eraclea all tested cultivars showed the highest positive scores, with the only exception of “Duprì” (score −0.006). The cultivars “Vettore”, “Gamenya”, “Italo”, “Simeto”, “Creso”, “Virgilio” and “Meridiano” had the highest scores (1.843, 1.839, 1.780, 1.724, 1.652, 1.615 and 1.526, respectively), suggesting these cultivars expressed their genetic background better in northern Italy than in the other two geographical areas. By contrast, the highest negative scores were observed at CIMMYT for the cultivars “Tresor”, “Italo”, “Vettore”, “Dylan”, “Creso”, “Tiziana” and “Gamenya” (−2.925, −2,498, −2.160, −1.711, −1.516, −1.474 and −1.436, respectively). The cultivar scores at Reggio Calabria were intermediate between the scores achieved at Eraclea and CIMMYT. 

## 3. Discussion

In Italy, since the 1970s, the cultivation of durum wheat, a traditional crop of southern and central regions, has expanded northward thanks to both the better productive performances of new cultivars in central and northern regions and the economic incentives of the European Union aimed at increasing the production of durum wheat for pasta making and reducing importations from third countries. However, in northern regions FHB may be a serious constraint for the cultivation of durum wheat as climatic conditions are favorable to infections by *Fusarium* species and accumulation of mycotoxins [45]. Consequently, FHB tolerance should be one of the main characteristics of durum wheat cultivars grown in northern Italy. 

Several studies in Italy addressed the genetics and physiology of the infection process of wheat by *F. graminearum,* the diversity of *Fusarium* populations associated with FHB of cereals, the regional distribution patterns of diverse *Fusarium* species, the toxins they produce and how the production of toxins is influenced by environmental conditions such as climate and soil management practices [2,9,45,46,47,48,49,50,51,52,53,54,55,56,57,58]. However, only for a restricted number of durum wheat cultivars was the susceptibility to FHB evaluated and, in some cases, only indirectly on the basis of mycotoxin content of their kernels [2,45,48,52,53]. In a previous study including eight durum wheat cultivars sampled in five distinct geographic areas from northern Italy to Sicily, “Simeto”, “Claudio” and “Duilio” showed a higher content of mycotoxins than “Dylan”, “Iride”, “Kanakis”, “Sculptur” and “Ramirez”, the last being the cultivar with the lowest content of mycotoxins [52]. However, Aureli et al. [52] did not correlate the mycotoxin contamination level of kernels to FHB field susceptibility and only four (“Duilio”, “Dylan”, “Iride” and “Simeto”) out of these eight cultivars were included in the test for FHB susceptibility at CIMMYT, Eraclea and Reggio Calabria. Therefore, it is difficult to make a comparison of results. However, consistently with the results of Aureli et al. [52], in field experiments at CIMMYT, Eraclea and Reggio Calabria, “Dylan” proved to be moderately resistant to FHB, while “Duilio” and “Simeto” were ranked as susceptible at all three sites. Conversely, at CIMMYT, Eraclea and Reggio Calabria the disease severity and FHB index values of “Iride” did not differ significantly from those of “Simeto” and “Duilio”. Quite interestingly, however, while the proportion of damaged kernels of “Simeto” and “Duilio” were much higher than the overall mean of all cultivars, thus confirming the extreme FHB susceptibility of these two cultivars, the proportion of damaged kernels for “Iride” was below the overall mean and very close to that of “Dylan”, suggesting the level of mycotoxin contamination is not necessarily correlated to FHB susceptibility. The susceptibility showed by “Simeto” is in agreement with a previous comparative study of other authors in Campania (southern Italy), including four durum wheat cultivars [2]. Inconsistencies between results of different studies may be, at least in part, imputed to differences in *Fusarium* populations associated with FHB in diverse cultivation areas [59]; this is why in the present study mixtures of *F. graminearum sensu latu* isolates sourced locally were used for inoculations of wheat cultivars. According to the literature, *F. graminearum* is the most common species of the *Fusarium* complex associated with FHB worldwide including central and southern Italy, while *F. culmorum* was reported to prevail over *F. graminearum* in Umbria (central Italy) and *F. poae* in some areas of northern and central Italy [2,45,46,53]. To make comparable results obtained in different environments, in this study the level of susceptibility of durum wheat cultivars, expressed in terms of FHB index, disease severity, disease incidence and proportion of infected kernels, was defined as relative to the susceptibility of standard bread wheat cultivars used internationally as references, such as the very susceptible “Gamenya” and “Sumai #3”, a highly resistant Chinese cultivar derived from a cross between the Italian cultivar “Funo” and the Chinese landrace “Taiwan Xiaomai” and recognized as the best source of FHB resistance worldwide [60]. Notwithstanding the fact that FHB is regarded as a threat to wheat cultivation in humid areas of northern and central Italy, very little information is available on the susceptibility of most durum wheat cultivars to this disease. The present study is the most comprehensive screening of Italian durum wheat cultivars for the susceptibility to FHB performed to date. Interestingly, the rankings of 16 Italian durum wheat cultivars based on FHB susceptibility at Eraclea (northern Italy), Reggio Calabria (southern Italy) and CIMMYT (Mexico) were very similar although the trials in Italy and in Mexico were carried out ten years apart from each other and in different environments. 

The PCA confirmed that in the years considered the tested cultivars performed consistently in the three different geographical areas with regards to the susceptibility to the disease. In general, cultivars grew better and showed a higher productive performance in northern Italy, while the grain quality in terms of protein content and thousand-kernels weight was better in southern Italy.

Interestingly, three medium to late-flowering cultivars, “Duprí”, “Dylan” and “Tiziana”, showed a very low FHB susceptibility in all three trials, although their resistance to the disease did not equal that of “Sumai #3”. Many durum wheat cultivars tested in this study, including “Plinio”, “Summa”, “Arcangelo”, “Daunia”, “Italo”, “Meridiano”, “Nibbio”, “Plinio”, “Saadi”, “Ulisse” and “Vettore”, turned out to be very susceptible, while most, including “Bravo”, “Campodoro”, “Creso”, “Crispiero”, “Duilio”, “Durango”, “Fortore”, “Gabbiano”, “Grecale”, “Iride”, “Levante”, “Nerone”, “Peleo”, “Perseo”, “Piceno”, “Poggio”, “Ramsete”, “Romano”, “Saragolla”, “Simeto”, “Tresor”, “Vettore” and “Virgilio”, were from susceptible to moderately susceptible. In all experiments the performance of bread wheat genotypes used as references, “Gamenya”, “Falcin”, “Heilo”, “Ocoroni F86”, “Sumai #3” and “Gondo/CBRD”, was in agreement with an internationally recognized FHB susceptibility scale that has been repeatedly validated in different cultivation areas worldwide [10,12,61]. This makes the results of the present screening of durum wheat cultivars for FHB susceptibility even more reliable. 

Within the group of cultivars tested, the susceptibility to the disease was inversely correlated with other phenotypic traits, i.e., HT and PH, indicating that, in general, early flowering and small-size durum wheat cultivars as well as those with a short crop cycle were more prone to FHB infections. In most trials for evaluating FHB resistance of wheat cultivars in the field, PH and FHB susceptibility were significantly correlated regardless of environmental conditions, with the result that taller plants were more FHB-resistant [62]. This has been explained assuming that PH is mainly a passive FHB resistance factor [62]. Crop debris on the soil surface act as an inoculum reservoir for primary infections and consequently heads of small-sized plants are more easily infected by ascospores and conidia dispersed by rain-splashes while tall plants escape the infection. However, a lower susceptibility of taller plants was found also when inoculum was applied from the top by spray inoculation, as in the trials performed in this study. It is very likely that PH may influence microclimate, including relative humidity, leaf wetness duration and temperature, at the ear level. Small-sized plants are more affected by soil humidity and dew and a dense canopy structure reduces air circulation. High humidity and warm temperature favor infection and disease development and may thus lead to an increase in disease pressure on small-sized plants [62]. Co-localization of QTL for FHB severity and PH has often been found, but it does not occur in all cases and the genetic basis of resistance in durum wheat is not fully understood [62]. Interestingly, the semi-dwarfing Rht1 alleles, governing plant height and widely used in modern wheat breeding, increase FHB susceptibility. Other genes, including the photoperiod insensitivity gene Ppd-D1a (2DS), the vernalization requirement genes Vrn-A1 (5AL) and Vrn-B1 (5BL) and major wheat domestication gene Q (5AL) were also found to be associated with PH and FHB resistance [62]. The correlation between FHB susceptibility and flowering-related traits of the wheat cultivars, such as HT and spike compactness, is not surprising as FHB is a floral disease. The phenotypic correlations and the genetic relationships between FHB, PH and flowering-related traits have been extensively investigated in bread wheat cultivars, mostly of Chinese origin [1,63,64]. In agreement with the results of the present study, including mostly durum wheat cultivars of Italian origin, also in bread wheat cultivars, there was found a strong inverse correlation between FHB susceptibility and HT. Although environmental conditions, such as temperature and humidity during anthesis, may influence FHB infection and disease development, a number of studies identified a genetic association between HT and FHB resistance [62]. 

Many durum wheat cultivars tested originally at CIMMYT in this study are now commercially available and some, such as “Iride”, “Saragolla” and “Simeto” are among the 10 most widely cultivated durum wheat cultivars in Italy. A few have been included in the official lists of durum wheat cultivars recommended by CREA (Agricultural Research Council and Economics, Rome, Italy) for northern and southern Italy (“Creso”, “Duilio”, “Dylan”, “Meridiano”, “Saragolla”, “Simeto” and “Tiziana”) and for central and northern Italy (“Levante” and “Virgilio”), respectively. Besides, “Iride”, “Levante” and “Saragolla” are reported in the 2020 catalog of Syngenta-Italy as FHB moderately tolerant. All the afore-mentioned cultivars are among those classified in this study as tolerant to moderately tolerant (“Dylan”, “Saragolla”, “Simeto” and “Virgilio”) or moderately susceptible (“Creso”, “Duilio” and “Iride”), with the only exception of “Meridiano” which in our tests proved to be very susceptible. However, as far as this last cultivar is concerned, it must be considered that in this study the evaluation of cultivars was performed under high disease pressure and the experimental conditions of the assays were very conducive to the infections.

An interesting finding of this study including a large number of durum wheat genotypes of Italian origin is the correlation between FHB susceptibility and some phenological and morphological traits of the cultivars, such as heading time and plant height. This suggests the possibility of using phenotypic characters as an aid in implementing breeding programs of durum wheat for FHB resistance. A recently published comprehensive review highlights the potential role of germplasm selection and morphological traits to improve FHB resistance in durum wheat [65]. Phenotypic selection for improved FHB resistance was over a long period the only option in resistance breeding and although during the past 20 years significant progress has been made in the knowledge of the genetic control of FHB resistance and wheat breeding for resistance to diseases [66,67,68] it still has several advantages over genomic selection when time and costs are not considered. Acquiring high-quality phenotypic data will certainly remain essential for further progress in resistance improvement. In agreement with our results, in a recent study aimed at evaluating an international collection of 228 durum wheat cultivars and at investigating their genetic background and the potential of genomic-assisted breeding for FHB resistance, a strong positive correlation between PH and FHB resistance was found and it was demonstrated that PH and QTL for FHB resistance are actually co-localized [69]. This encourages us to pursue the objective of exploring further and exploiting the Italian wheat germplasm to enhance FHB resistance of commercial durum wheat cultivars with desirable agronomic traits.

## 4. Materials and Methods 

### 4.1. Wheat Genotypes 

Overall, 35 cultivars of durum wheat [*Triticum turgidum* L. subsp. *durum* (Desf.) Husn.] of Italian origin, supplied by Italian public research institutions and private companies, were tested. Six genotypes of bread wheat (*Triticum aestivum* L. subsp. aestivum) were included as references: “Sumai #3” and “Gondo/CBRD” (FHB resistant), “Heilo” (FHB moderately resistant), “Ocoroni F86” (FHB moderately susceptible), “Gamenya” and “Falcin” (FHB susceptible) [10,12,60,61]. 

Kernels of all tested cultivars were deposited in the germplasm bank of CIMMYT (International Maize and Wheat Improvement Center, El Batán, Texcoco, Mexico). Moreover, part of durum wheat cultivars and the two bread wheats “Sumai #3” and “Gamenya” were deposited in the germplasm bank of CREA (Agricultural Research Council and Economics–Research Centre for Cereal and Industrial Crops, Acireale, Catania, Italy).

All 35 durum wheat cultivars and six genotypes of bread wheat used as references were included in the trial performed in 2009 at CIMMYT in Mexico (Table 1). 

Only a selection of 16 of these 35 durum wheat cultivars and two genotypes of bread wheat, used as references, (“Sumai #3” and “Gamenya”) were included in the trials performed in the 2019–2020 cropping season in Italy (Table 3). All the 16 cultivars were selected as they are included in the official lists of durum wheat cultivars of CREA while the rest of cultivars tested at CIMMYT is not longer on these lists.

### 4.2. Inoculum 

Inoculum was a mixture of five mono-conidial isolates of *F. graminearum sensu lato* selected out of a larger number (about 70) of isolates for both their virulence and ability to produce DON on rice medium, in accordance with a standard protocol used routinely at CIMMYT [12]. For the trial performed at CIMMYT the isolates were obtained from naturally infected spikes collected in different commercial farms in Mexico the year before, to ensure the viability and virulence of the pathogen, while for the trial performed at Reggio Calabria the isolates were obtained from naturally infected spikes collected the year before in different commercial farms in Lombardy and Emilia Romagna (northern Italy). 

To obtain mono-conidial isolates, distilled water (9 mL) was pipetted into a glass tube and sterilized in autoclave. Single conidia were taken from Petri dishes containing SNA (Spezieller Nahrstoffarmer Agar, Innovation GmbH, Köln, Germany) and put into glass tubes. The excess of mycelium was eliminated, the tubes were shaken and left to rest for about 10 min. The content of the glass tubes was then transferred to Petri dishes on agar–water (20 g of agar in 1 L of distilled water, autoclaved at 120 °C for 15 min). The water excess was removed, the dishes were left to dry and then incubated for one day at 25 °C. The agar-water dishes incubated as previously described were observed at the stereomicroscope and single conidia were picked up using a needle and transferred to Petri dishes containing SNA. Dishes were incubated for seven days at 25 °C in the dark. Mono-conidial isolates recovered from infected spikes were identified as *F. graminearum sensu lato* on the basis of their morphological characteristics on CLA (Carnation Leaf Agar) [70,71,72] and by DNA PCR amplification using the specific primer set FG16 N F/R [73]. Subsequently, the TOXP1/2 primer set was used for chemotype classification [49]. The ability of isolates to produce DON was verified following a standard protocol [74]. An Erlenmeyer flask (300 mL) containing dry polished rice (30 g) and distilled water (d.w.) (15 mL) were autoclaved at 120 °C for 20 min and inoculated with 2–3 mycelium plugs. After inoculation, flasks were shaken for two days and incubated at 25 °C in the dark. After 14 days incubation, the rice medium was freeze-dried in a lyophilizer and ground with a coffee grinder. Two grams of ground sample were put into a 50 mL tube, 40 mL of d.w. were added and the sample was shaken for 2 min. An aliquot of 400 µL was put into a 2 mL tube and 1.6 mL of d.w. were added (5 times dilution). After centrifugation (14,000 rpm for 20 minutes) 50 µL of supernatant were taken using a pipette and tested by an ELISA kit (RIDASCREEN® FAST DON, R-Biopharm AG, Darmstadt, Germany). The virulence of isolates was tested in greenhouse on two resistant, Sumai #3 and Heilo and three susceptible wheat genotypes, “SERI/CEP80120”, “BCN//DOY1/*Aegilops squarrosa*” (447) and “Gamenya” using the standard syringe inoculation method [75,76,77]. To inoculate the wheat plants at CIMMYT the four highest ranked isolates were selected and mixed with a control isolate with known aggressiveness selected previously and used at CIMMYT in the inoculum mixture of the previous year, to generate the new inoculum for the year”s field screening. The inoculum mixture used in Reggio Calabria and Eraclea was a mixture of five selected, highly virulent isolates collected the year before the test, all of Italian origin. 

### 4.3. Inoculum Production for Artificial Inoculations of Wheat Cultivars in the Field

Five to six agar plugs of monosporic *F. graminearum* cultures were used to inoculate 500 mL Erlenmeyer flasks containing 200 mL of Lima bean (*Phaseolus lunatus* L.) broth [78]. The medium was prepared with 20 g L^−1^ of dried Lima beans in 1 L of d.w.; beans were washed thoroughly, covered with d.w. and boiled until the color of the water turned red. The liquid was filtered, adjusted to 1 L volume and autoclaved at 120 °C for 20 min. Flasks were incubated in a horizontal stirrer at 200 rpm for 7 days at room temperature (22–25 °C). After 7 days incubation, the cultures were filtered, poured into a 250 mL flask and stored at 4 °C to allow sedimentation. After complete sedimentation, the liquid was centrifuged at 3000 rpm for 10 min, the surnatant was discarded and the pellet containing conidia was re-suspended in sterile d.w. A 0.5 mL volume of the suspension was poured into 100 mL of sterilized distilled water (s.d.w.) to obtain a final concentration of 1 × 105 conidia mL^−1^. Aliquots (100 µL) of the suspension were pipetted into Petri dishes containing Lima bean agar medium. The suspension was spread thoroughly on the surface of the medium and dishes were then incubated at 25 °C for 7 days with a 12 h photoperiod. After incubation, 40 dishes were superficially scraped and the mycelium was poured into 2 L of s.d.w. The suspension, containing mainly conidia, was diluted with s.d.w. up to a concentration of 5 × 10^4^ conidia mL^−1^. Conidial concentration was adjusted with a Neubauer-counting chamber.

### 4.4. Field Experiments

This study included three distinct experiments carried out in different environments and climatic areas, one performed in 2009 in Mexico and the other two performed in 2019–2020 in northern and southern Italy, respectively. The first field test was carried out in 2009 at CIMMYT”s headquarters, close to Mexico City (altitude of 2240 m a.s.l., with an average annual precipitation of 625 mm and an average annual temperature of about 16 °C). The rainfall of the period first June–15 October was 222 mm. The minimum temperature varied from 9 °C in the first decade of June to 15 °C in the third decade of July and August, while the maximum temperature varied from 22 °C in the third decade of August and the first and second decade of September at 28 °C in the first ten days of June. Relative humidity varied from 58% in the first decade of June to 85% in the first and third decade of September. The field test included all the 35 durum wheat cultivars and the six bread wheat genotypes used as references. Wheat genotypes were sown during the first week of June 2009 (summer cycle, with spring sowing to have a second harvest) at the El Batàn Station on 1 m double rowed plots, where maize had been the previous crop. The experimental design was a randomized complete block design with two replications. Sowing was performed by means of a sowing machine, using 50 g of seed for each plot. Plots were irrigated soon after the sowing, to favor a fast and homogeneous germination. Nitrogen (150 kg ha^−1^) and phosphorous (40 kg ha^−1^) were applied twice, soon after the sowing and 40 days later. The entire experimental field was equipped with a fine misting system in order to maintain high air moisture conditions, which are requested for FHB infection and development after the inoculation. Misting was ensured by DAN modular micro-sprinklers, arranged in a 3 × 4 m scheme. The system was managed by a programmable timer to ensure high (around 100%) moisture conditions 24 h a day. In each plot, inoculation with the conidia suspension (5 × 10^4^ mL^−1^) was performed when at least 50% of the plants were in the full flowering stage, using a CO_2_ sprayer (3 s per plot). The inoculation was repeated 2 days later.

Two additional distinct field tests were carried out 10 years later in 2019–20 cropping season in Italy, at the Mediterranean University of Reggio Calabria, close to the town of Reggio Calabria (southern Italy) (altitude 235 meters a.s.l., with an average annual precipitation of 803 mm and an average annual temperature of about 18.3 °C) and in a commercial farm at Eraclea, close to the town of Venice in northern Italy (altitude 5 meters a.s.l., with an average annual precipitation of 810 mm and an average annual temperature of about 12.7 °C). Both trials included 16 durum wheat cultivars evaluated previously at CIMMYT and included in the Italian National Plant Variety Register and the two reference genotypes of bread wheat “Sumai #3” and “Gamenya”.

Wheat genotypes were sown during the second week of December 2019 at the experimental field of the Mediterranean University of Reggio Calabria and during the second week of January 2020 at the experimental field of Eraclea, in plots of 2.5 m^2^ (1 m × 2.5 m). Naked fallow and soybean had been the previous crop in the first and in the second field, respectively. The experimental design, in both field tests, was a randomized complete block design with three replications. Sowing was performed by means of a sowing machine, using 50 g of seed for each plot. Nitrogen (36 and 18 kg ha^−1^, respectively) and phosphorous (92 and 46 kg ha^−1^) were applied soon after the sowing. An additional dose of 46 kg ha^−1^ of nitrogen was applied in late February and in late March, respectively. 

To keep air moisture high and ensure favorable conditions for FHB infections, the experimental fields were equipped with a misting irrigation system, with micro-sprinklers arranged in a 3 × 4 m scheme. The system was managed by a programmable timer to ensure high (around 100%) moisture conditions 24 h a day. In each plot, inoculation with the conidia suspension (5 × 10^4^ mL^−1^) was performed when at least 50% of the plants were in the full flowering stage, using a CO_2_ sprayer (3 s per plot). The inoculation was repeated 2 days later. 

### 4.5. Disease Score and Evaluation of Morpho-Physiological Characteristics of Wheat Genotypes

In all three field trials symptoms were evaluated by visual inspection 30 days after inoculation.

For each genotype, 10 plants per plot were chosen at random. The disease was scored using the FHB index, which was calculated as follows: FHB index = disease severity * disease incidence/100, where disease severity was the number of symptomatic spikelets/total spikelets * 100 and disease incidence was the number of symptomatic spikes/total spikes * 100 [79]. To confirm the visual score, 10 seeds per plot from symptomatic spikelets were put in Petri dishes on water-agar, incubated at 25 °C for one day and examined under the stereomicroscope to detect the presence of typical *Fusarium* conidia. In the field test set up in Mexico, heading time and physiological ripening (both expressed as time intervals, in days, after 1st August) and plant height (measured in cm) were determined for each plot. After harvesting, carried out in mid-October of the same year, the thousand-kernels weight (TKW) and the number and weight of seeds per spike were determined.

In the field tests set up in Italy, heading time and physiological ripening (both expressed as time intervals, in days, after 1st April) and plant height (PH), in cm, were determined for each plot. After harvesting, carried out on 15th June 2020 at Reggio Calabria and on 15th July 2020 at Eraclea, the grain yield (GY), TKW, hectolitre weight (HW) and protein content (PC) were determined.

### 4.6. Statistical Analysis

Statistical analysis of data was performed using the Statgraphics® Centurion XVI software package (Statpoint Technologies, INC.). A one-way analysis of variance (ANOVA), followed by Multiple Range Test of Student-Newman-Keul (SNK) (* *p* ≤ 0.05), was carried out on data from the trial at CIMMYT in Mexico. Factorial analysis of variance (factorial ANOVA) followed by Multiple Range Test of Student–Newman–Keul (SNK) test (*** *p* ≤ 0.001) was carried out on data from the two trials performed in Italy. The data were expressed as means ± standard deviations. The comparison among the three locations was carried out through the Principal Component Analysis (PCA) of the average data using the PAST, PAleontological STatistics software package, 2011 [80], in order to graphically summarize the specific responses of the tested CVs and to highlight any similarities or dissimilarities.

## 5. Conclusions

The results of this screening are the only available information on the FHB susceptibility of many Italian durum wheat cultivars based on experimental data under high disease pressure and controlled experimental conditions. In agreement with previous studies highlighting the difficulty in finding germplasm resistant to FHB in international collections of durum wheat germplasm [40,44,54], very few of the Italian durum wheat cultivars tested proved to be resistant to this disease, whereas most of them were ranked from moderately susceptible to very susceptible. This finding indicates that, at present, in an integrated disease management strategy, including chemical treatments, when appropriate, the use of tolerant/resistant or moderately susceptible cultivars as well as the reduction of initial amount of inoculum by correct agronomic techniques, such as crop rotation avoiding corn as a previous crop, time of sowing, stubble burning and deep tillage are the most effective options to mitigate the impact of FHB. The results of this study provide valuable information on FHB susceptibility of available durum wheat cultivars in Italy and may be useful to guide farmers in choosing the cultivars most suitable for areas in which environmental conditions are favorable to this disease.

Moreover, the information on FHB susceptibility of durum wheat germplasm could be successfully utilized in breeding programs for FHB resistance. In this respect, it seems interesting to note the strong correlation that was found between FHB susceptibility and other phenotypic traits, such as PH and HT, which is in agreement with recent findings of breeders involved in the genetic improvement of durum wheat [62,65,79]. 

In general, a preliminary comprehensive screening of local germplasm in controlled environmental conditions and diverse environments is the basis for any breeding program for FHB resistance pursued using conventional or modern molecular methods. 

## Figures and Tables

**Table 1:** Susceptibility to Fusarium head blight (FHB) of 35 Italian durum wheat cultivars and six bread wheat cultivars used as references, artificially inoculated with *Fusarium graminearum*, as evaluated at CIMMYT, Mexico and expressed in terms of proportion of damaged kernels, disease severity, disease incidence and FHB index ^1^.

Cultivars	Damaged Kernels [%]	Disease Severity [%]	Disease Incidence [%]	FHB Index [%]
ARCANGELO	14.1 ± 1.56 b	39.3 ± 2.26 a–e	97.5 ± 3.54 a	38.3 ± 0.49 a–e
BRAVO	16.5 ± 3.89 b	25.9 ± 3.96 c–g	90.0 ± 14.14 ab	23.3 ± 5.59 c–g
CAMPODORO	16.2 ± 1.13 b	22.4 ± 0.35 d–h	95.0 ± 7.07 a	21.3 ± 0.35 d–h
CRESO	13.7 ± 0.28 b	37.0 ± 1.63 b–e	100.0 ± 0.00 a	37.0 ± 1.63 b–e
CRISPIERO	15.9 ± 0.28 b	27.9 ± 4.67 c–g	97.5 ± 3.54 a	27.2 ± 5.66 c–f
DAUNIA	20.5 ± 1.98 b	40.1 ± 2.55 a–d	100.0 ± 0.00 a	40.1 ± 2.55 a–d
DUILIO	18.9 ± 5.52 b	36.0 ± 4.03 b–e	97.5 ± 3.54 a	35.1 ± 5.37 b–e
DUPRÍ	12.0 ± 3.32 b	9.4 ± 7.00 h–i	65.0 ± 28.28 bc	6.1 ± 3.96 hi
DURANGO	17.3 ± 0.14 b	31.1 ± 0.78 b–g	90.0 ± 14.14 ab	28.0 ± 0.64 b–f
DYLAN	10.5 ± 1.98 b	15.6 ± 7.85 f-i	67.5 ± 3.54 a–c	10.5 ± 6.01 f–i
FORTORE	18.5 ± 2.12 b	33.1 ± 7.92 b–f	97.5 ± 3.54 a	32.4 ± 8.98 b–e
GABBIANO	17.1 ± 10.04 b	26.3 ± 0.21 c–g	97.5 ± 3.54 a	25.6 ± 0.85 c–g
GRECALE	20.9 ± 0.49 b	28.6 ± 1.48 b–g	97.5 ± 3.54 a	27.9 ± 0.28 b–f
IRIDE	14.3 ± 2.69 b	34.5 ± 15.70 b–e	100.0 ± 0.00 a	34.5 ±15.70 b–e
ITALO	18.0 ± 1.63 b	41.0 ± 5.02 a–d	100.0 ± 0.00 a	41.0 ± 5.02 a–d
LEVANTE	12.8 ± 3.25 b	26.3 ± 7.85 c–g	90.0 ± 14.14 ab	23.7 ± 9.05 c–g
MERIDIANO	14.1 ± 4.24 b	40.8 ± 6.93 a-d	100.0 ± 0.00 a	40.8 ± 6.93 a–d
NERONE	16.7 ± 0.07 b	23.1 ± 3.82 c–h	87.5 ± 3.54 ab	20.2 ± 4.17 e–h
NIBBIO	13.5 ± 10.39 b	41.2 ± 0.28 a–d	100.0 ± 0.00 a	41.2 ± 0.28 a–d
PELEO	19.7 ± 3.39 b	33.4 ± 0.99 b–f	100.0 ± 0.00 a	33.4 ± 0.99 b–e
PERSEO	18.2 ± 7.99 b	33.9 ± 6.93 b–f	100.0 ± 0.00 a	33.9 ± 6.93 b–e
PICENO	17.8 ± 2.33 b	27.4 ± 9.48 c–g	92.5 ± 10.61 a	25.3 ± 12.87 c–g
PLINIO	20.6 ± 10.47 b	46.9 ± 2.40 ab	100.0 ± 0.00 a	46.9 ± 2.40 ab
POGGIO	20.4 ± 3.54 b	30.2 ± 10.96 b–g	95.0 ± 7.07 a	28.7 ± 12.73 b–f
RAMSETE	15.0 ± 9.12 b	36.0 ± 1.98 b–e	97.5 ± 3.54 a	35.1 ± 3.61 b–e
ROMANO	19.3 ± 3.39 b	20.5 ± 1.41 e–h	95.0 ± 7.07 a	19.5 ± 2.97 e–h
SAADI	20.9 ± 6.72 b	42.5 ± 3.89 a–c	100.0 ± 0.00 a	42.5 ± 3.89 a–c
SARAGOLLA	14.0 ± 1.98 b	23.1 ± 6.43 c–h	92.5 ± 10.61 a	21.4 ± 8.63 d–h
SIMETO	20.3 ± 0.49 b	27.8 ± 3.25 c–g	100.0 ± 0.00 a	27.8 ± 3.25 b–f
SUMMA	32.2 ± 1.41 a	46.8 ± 4.24 ab	100.0 ± 0.00 a	46.8 ± 4.24 ab
TIZIANA	12.0 ± 1.20 b	13.5 ± 0.42 g–i	67.5 ± 17.68 a–c	9.1 ± 0.99 g–i
TRESOR	16.7 ± 4.53 b	29.8 ± 15.84 b–g	97.5 ± 3.54 a	29.1 ± 16.55 b–e
ULISSE	14.7 ± 1.41 b	42.0 ± 0.49 a–d	100.0 ± 0.00 a	42.0 ± 0.49 a–c
VETTORE	17.6 ± 6.36 b	40.0 ± 5.73 a–d	100.0 ± 0.00 a	40.0 ± 5.73 a–d
VIRGILIO	17.6 ± 1.06 b	25.0 ± 1.41 c–g	92.5 ± 10.61 a	23.1 ± 2.33 c–g
FALCIN	18.5 ± 4.10 b	24.7 ± 8.06 c–g	97.5 ± 3.54 a	24.1 ± 9.48 c–g
GAMENYA	22.3 ± 1.41 b	54.5 ± 6.08 a	100.0 ± 0.00 a	54.5 ± 6.08 a
GONDO/CBRD	4.9 ± 2.69 c	3.6 ± 1.98 i	55.0 ± 14.14 c	2.0 ± 1.34 i
HEILO	5.0 ± 1.13 c	27.9 ± 6.93 c–g	97.5 ± 3.54 a	27.2 ± 7.85 c–f
OCORONI F 86	12.6 ± 4.24 b	31.4 ± 3.11 b–g	100.0 ± 0.00 a	31.4 ± 3.11 b–e
SUMAI #3	3.9 ± 0.28 c	2.1 ± 1.48 i	35.0 ± 14.14 d	0.7 ± 0.49 i

^1^ Data are means of two replicates ±SD. Data in the same column followed by different letters are significantly different (Student–Newman–Keul test, *p* ≤ 0.05). Statistical analysis was performed after angular transformation of data.

**Table 2:** Phenological and grain traits of 35 Italian durum wheat cultivars and 6 bread wheat cultivars used as references, tested at International Maize and Wheat Improvement Center (CIMMYT), Mexico, for susceptibility to Fusarium head blight (FHB) ^1^.

Cultivars	Heading Time (days from 01/08)	Plant Height (cm)	Physiological Maturity (days from 01/08)	Seeds Per Spike (No)	Thousand Kernels Weight (g)
ARCANGELO	14.0 ± 4.24 f–h	54.5 ± 0.71 f–i	71.5 ± 3.54 a–c	25.3 ± 0.78 b–e	28.4 ± 5.02 ab
BRAVO	31.0 ± 0.00 a	73.5 ± 4.95 c–e	69.5 ± 6.36 a–d	20.2 ± 5.80 d–e	30.5 ± 1.91 ab
CAMPODORO	25.0 ± 1.41 a–d	73.0 ± 2.83 c–e	72.5 ± 2.83 ab	25.8 ± 1.56 b–e	33.1 ± 6.65 ab
CRESO	17.0 ± 0.00 e–h	55.0 ± 1.41 f–i	62.0 ± 0.00 b–e	23.1 ± 0.99 b–e	33.9 ± 1.06 a
CRISPIERO	20.5 ± 4.95 c–g	69.0 ± 7.07 c–h	69.5 ± 6.36 a–d	19.5 ± 10.96 d–e	38.3 ± 1.70 a
DAUNIA	11.0 ± 0.00 h	60.0 ± 1.41 e–i	68.5 ± 7.78 a–d	23.6 ± 1.56 b–e	26.0 ± 4.81 ab
DUILIO	17.0 ± 0.00 e–h	68.0 ± 5.66 c–h	65.0 ± 0.00 a–e	30.7 ± 5.37 b–e	34.6 ± 0.21 a
DUPRÍ	25.5 ± 2.12 a–d	65.0 ± 0.00 d–h	74.0 ± 0.00 a	31.8 ± 0.92 b–e	32.7 ± 1.77 a
DURANGO	31.0 ± 0.00 a	80.0 ± 0.00 cd	74.0 ± 0.00 a	25.1 ± 0.35 b–e	31.1 ± 5.87 ab
DYLAN	22.0 ± 2.83 b–f	62.5 ± 0.71 e–i	65.0 ± 0.00 a–e	35.6 ± 6.15 b–e	36.0 ± 2.83 a
FORTORE	18.5 ± 2.12 d–h	67.5 ± 4.95 c–h	65.0 ± 0.00 a–e	25.9 ± 4.95 b–e	27.2 ± 3.32 ab
GABBIANO	24.0 ± 0.00 a–e	72.5 ± 3.54 c–f	69.5 ± 6.36 a–d	19.0 ± 3.46 d–e	31.5 ± 2.55 a
GRECALE	14.0 ± 0.00 f–h	66.5 ± 4.95 d–h	65.5 ± 0.71 a–e	30.9 ± 5.66 b–e	26.8 ± 4.45 ab
IRIDE	18.5 ± 2.12 d–h	62.5 ± 10.61e–i	65.0 ± 12.73 a–e	36.6 ± 0.99 b–e	32.6 ± 0.35 a
ITALO	17.0 ± 0.00 e–h	52.5 ± 3.54 hi	56.0 ± 0.00 e	32.6 ± 3.96 b–e	25.3 ± 3.46 ab
LEVANTE	20.5 ± 4.95 cg	65.5 ± 7.78 d–h	74.0 ± 0.00 a	21.8 ± 7.85 d–e	32.7 ± 0.35 a
MERIDIANO	17.0 ± 0.00 eh	70.5 ± 4.24 c–g	68.0 ± 4.24 a–d	29.2 ± 2.83 b–e	37.6 ± 2.19 a
NERONE	25.0 ± 1.41 a–d	80.0 ± 4.24 cd	74.0 ± 0.00 a	26.6 ± 7.99 b–e	27.7 ± 7.57 ab
NIBBIO	17.0 ± 0.00 e–h	64.0 ± 8.49 d–h	56.0 ± 0.00 e	27.4 ± 1.63 b–e	25.9 ± 1.98 ab
PELEO	15.5 ± 2.12 f–h	63.0 ± 2.83 d–i	60.5 ± 6.36 c–e	34.9 ± 1.48 b–e	33.4 ± 6.58 a
PERSEO	14.0 ± 4.24 f–h	63.5 ± 4.95 d–i	60.5 ± 6.36 c–e	32.2 ± 5.66 b–e	35.5 ± 2.19 a
PICENO	20.5 ± 4.95 c–g	66.0 ± 0.00 d–h	63.5 ± 2.12 a–e	21.1 ± 11.38 d–e	33.7 ± 8.56 a
PLINIO	17.0 ± 0.00 e–h	61.5 ± 2.12 e–i	69.5 ± 6.36 a–d	25.3 ± 0.71 b–e	30.6 ± 7.07 ab
POGGIO	15.5 ± 2.12 f–h	61.0 ± 1.41 e–i	65.0 ± 0.00 a–e	31.0 ± 2.76 b–e	37.3 ± 5.16 a
RAMSETE	11.0 ± 0.00 h	58.5 ± 0.71 e–i	56.0 ± 0.00 e	30.7 ± 2.12 b–e	34.9 ± 8.20 a
ROMANO	27.5 ± 4.95 a–c	69.0 ± 1.41 c–h	74.0 ± 0.00 a	25.5 ± 2.33 b–e	33.6 ± 0.14 a
SAADI	17.0 ± 0.00 e–h	63.5 ± 9.19 d–i	69.5 ± 6.36 ad	25.7 ± 6.08 be	33.2 ± 0.35 a
SARAGOLLA	24.0± 0.00 a–e	75.5 ± 2.12 c–e	74.0 ± 0.00 a	25.3 ± 5.59 be	35.5 ± 4.74 a
SIMETO	28.5 ± 3.54 ab	62.5 ± 6.36 e–i	74.0 ± 0.00 a	15.8 ± 7.00 e	29.1 ± 1.77 ab
SUMMA	11.0 ± 0.00 h	65.0 ± 1.41 d–h	60.5 ± 6.36 c–e	16.4 ± 4.10 e	29.0 ± 3.89 ab
TIZIANA	22.0 ± 2.83 d–f	64.5 ± 2.12 d–h	65.0 ± 0.00 a–e	40.4 ± 0.35 ad	38.4 ± 0.49 a
TRESOR	18.5 ± 2.12 d–h	47.5 ± 3.54 i	56.0 ± 0.00 e	22.4 ± 3.25 ce	27.1 ± 2.47 ab
ULISSE	17.0 ± 0.00 e–h	65.5 ± 0.71 d–h	65.0 ± 0.00 a–e	24.5 ± 1.91 be	28.5 ± 0.78 ab
VETTORE	15.5 ± 2.12 f–h	61.5 ± 0.71 e–i	56.0 ± 0.00 e	31.3 ± 8.13 be	25.9 ± 0.92 ab
VIRGILIO	25.0 ± 1.41 a–d	68.0 ± 2.83 ch	69.5 ± 6.36 ad	18.6 ± 2.47 de	27.2 ± 0.42 ab
FALCIN	11.0 ± 0.00 h	70.5 ± 0.71 c–g	60.5 ± 6.36 c–e	43.7 ± 3.04 a–c	37.4 ± 1.91 a
GAMENYA	17.0 ± 0.00 eh	82.5 ± 3.54 c	56.0 ± 0.00 e	19.9 ± 0.92 d–e	16.5 ± 1.77 b
GONDO/CBRD	25.5 ± 2.12 a–d	91.5 ± 2.12 b	72.5 ± 2.12 ab	54.4 ± 13.36 a	37.3 ± 2.33 a
HEILO	12.5 ± 2.12 g–h	62.5 ± 3.54 e–i	65.0 ± 12.73 a–e	38.4 ± 1.41 b–d	28.1 ± 3.18 b
OCORONI F 86	11.0 ± 0.00 h	62.0 ± 7.07 e–i	59.0 ± 4.24 d–e	40.0 ± 0.07 a–d	28.0 ± 3.04 ab
SUMAI #3	26.0 ± 0.00 a–d	105.0 ± 7.07 a	74.0 ± 0.00 a	44.0 ± 6.22 ab	33.1 ± 2.90 a

^1^ Data are means of two replicates ±SD. Data in the same column followed by different letters are significantly different (Student–Newman–Keul test, *p* ≤ 0.05).

**Table 3:** Factorial ANOVA of the disease susceptibility indices and some morpho-physiological traits of 16 Italian durum wheat cultivars and 2 reference cultivars of bread wheat evaluated for their susceptibility to Fusarium head blight (FHB) ^1^. The test was performed in two different sites in northern and southern Italy (Eraclea, Veneto region and Reggio Calabria, Calabria region, respectively).

Factors of Variability	Site	Cultivar	N^1^	Damaged Kernels (%)	Disease Severity (%)	Disease Incidence (%)	FHB Index (%)	Heading Time ( (days from 01/04)	Plant Height (cm)
**Site (A)**				***	***	**	***	***	***
	Eraclea		54	18.3 ± 5.03	32.8 ± 13.38	91.9 ± 15.3	31.9 ± 14.56	30.6 ± 5.45	87.3 ± 11.92
	RC		54	16.8 ± 4.73	29.6 ± 13.15	89.6 ± 16.37	27.8 ± 13.55	25.9 ± 5.11	83.8 ± 11.89
**Cultivar (B)**				***	***	***	***	***	***
		CRESO	6	14.1 ± 0.52 fg	37.0 ± 0.92 cd	98.3 ± 2.58 a	36.4 ± 1.35 cd	31.7 ± 3.33 c	76.5 ± 1.87 hi
		DUILIO	6	21.2 ± 0.97 b	36.7 ± 1.65 cd	100.0 ± 0.00 a	36.7 ± 1.65 cd	23.3 ± 2.25 hi	82.7 ± 2.16 e–g
		DUPRI”	6	15.6 ± 0.66 b	11.4 ± 1.42 h	68.8 ± 5.85 c	7.8 ± 1.31 g	29.2 ± 3.97 de	76.3 ± 3.98 i
		DYLAN	6	13.0 ± 0.57 fg	14.0 ± 0.83 h	70.5 ± 3.94 c	9.9 ± 0.77 g	30.5 ± 3.62 cd	82.2 ± 2.04 e–g
		FORTORE	6	19.2 ± 1.25 b	36.0 ± 4.34 cd	100.0 ± 0.00 a	36.0 ± 4.34 cd	25.3 ± 2.66 g	90.0 ± 2.28 c
		GRECALE	6	21.5 ± 1.09 g	28.9 ± 0.89 ef	100.0 ± 0.00 a	28.9 ± 0.89 ef	25.2 ± 2.40 gh	79.7 ± 1.03 f–h
		IRIDE	6	16.0 ± 2.27 e	41.4 ± 5.43 bc	100.0 ± 0.00 a	41.4 ± 5.43 bc	25.3 ± 2.94 g	77.2 ± 2.04 hi
		ITALO	6	21.8 ± 0.82 bc	40.1 ± 1.13 bc	100.0 ± 0.00 a	33.6 ± 7.74 de	22.0 ± 1.90 il	85.0 ± 4.15 de
		MERIDIANO	6	17.3 ± 1.12 e	39.7 ± 1.38 bc	99.2 ± 2.04 a	39.4 ± 2.00 b–d	31.8 ± 3.19 c	83.0 ± 3.16 ef
		SAADI	6	22.4 ± 0.50 h	43.1 ± 2.01 b	100.0 ± 0.00 a	43.1 ± 2.01 b	25.0 ± 1.79 gh	76.2 ± 3.31 i
		SARAGOLLA	6	14.0 ± 0.98 a	25.8 ± 4.13 g	95.8 ± 6.65 a	24.7 ± 4.38 f	25.7 ± 2.25 g	80.7 ± 1.75 fg
		SIMETO	6	21.4 ± 1.77 ef	33.4 ± 4.74 de	100.0 ± 0.00 a	33.4 ± 4.74 de	24.7 ± 2.58 gh	79.3 ± 1.37 g–i
		TIZIANA	6	12.4 ± 0.96 g	12.9 ± 0.96 h	67.4 ± 5.04 b	10.6 ± 1.65 g	34.8 ± 2.93 b	81.0 ± 1.41 fg
		TRESOR	6	17.1 ± 2.21 cd	36.7 ± 5.32 cd	99.2 ± 2.04 a	36.3 ± 5.05 cd	21.3 ± 2.66 l	82.3 ± 4.50 e–g
		VETTORE	6	20.4 ± 1.45 b	38.8 ± 1.43 b.d	95.8 ± 6.65 a	37.2 ± 3.25 cd	27.7 ± 2.16 ef	90.0 ± 2.61 c
		VIRGILIO	6	17.3±1.15 ef	29.5 ± 6.25 ef	92.5 ± 7.58 a	27.5 ± 6.91 f	26.3 ± 2.25 fg	87.7 ± 1.03 cd
		SUMAI #3	6	4.80 ± 0.34 b	2.2 ± 0.48 i	47.5 ± 5.24 d	1.1 ± 0.29 h	38.7 ± 2.58 a	125.2 ± 2.13 a
		GAMENYA	6	25.9 ± 1.49 de	54.2 ± 1.61 a	100.0 ± 0.00 a	54.2 ± 1.61 a	39.5 ± 2.43 a	104.5 ± 2.53 b
**A x B**				***	***	***	***	***	***
	Eraclea	CRESO	3	14.5 ± 0.35 l–o	37.2 ± 0.82 b–f	100.0 ± 0.00 a	37.2 ± 0.8 2 b–g	34.7 ± 0.58 de	78.0 ± 1.00 l–n
		DUILIO	3	22.1 ± 0.26 b–d	38.2 ± 0.55 b–e	100.0 ± 0.00 a	38.2 ± 0.55 b–f	25.3 ± 0.58 i–o	84.3 ± 1.53 e–i
		DUPRI”	3	16.0 ± 0.25 h–m	12.4 ± 0.95 h	71.0 ± 3.61 b	8.8 ± 0.98 m	32.7 ± 0.58 e	79.7 ± 1.53 i–n
		DYLAN	3	13.5 ± 0.35 l–o	14.4 ± 0.61 h	71.0 ± 3.61 b	10.2 ± 0.69 m	33.7 ± 1.53 e	83.0 ± 2.65 f–m
		FORTORE	3	19.9 ± 0.36 c–f	37.9 ± 1.31 b–e	100.0 ± 0.00 a	37.9 ± 1.31 b–f	27.7 ± 0.58 f–i	91.7 ± 1.53 d
		GRECALE	3	22.4 ± 0.35 b–d	29.4 ± 0.45 e–g	100.0 ± 0.00 a	29.4 ± 0.45 f–i	27.3 ± 0.58 f–i	79.7 ± 0.58 i–n
		IRIDE	3	17.7 ± 0.66 f–i	44.6 ± 0.79 b	100.0 ±0.00 a	44.6 ± 0.79 bc	28.0 ± 0.00 f–i	79.0 ± 0.00 i–n
		ITALO	3	22.1 ± 0.36 b–d	40.3 ± 0.55 bc	100.0 ± 0.00 a	40.3 ± 0.55 b–d	23.7 ± 0.58 m–q	88.7 ± 0.58 de
		MERIDIANO	3	17.9 ± 0.57 f–i	40.7 ± 0.80 bc	100.0 ± 0.00 a	40.7 ± 0.80 b–d	34.7 ± 0.58 b–e	85.3 ± 1.53 e–h
		SAADI	3	22.7 ± 0.36 bc	44.8 ± 0.95 b	100.0 ± 0.00 a	44.8 ± 0.95 b	26.3 ± 0.58 f–m	79.0 ± 1.00 i–n
		SARAGOLLA	3	14.1 ± 0.6 l–o	28.3 ± 1.6 fg	100.0 ± 0.00 a	28.3 ± 1.60 g–l	27.7 ± 0.58 f–i	80.7 ± 1.15 h–m
		SIMETO	3	23.0 ± 0.15 bc	35.9 ± 1.76 b–f	100.0 ± 0.00 a	35.9 ± 1.76 b–g	27.0 ± 0.00 f–l	80.0 ± 1.00 i–n
		TIZIANA	3	13.0 ± 0.31 m–o	13.6 ± 0.70 h	67.7 ± 2.52 b	9.2 ± 0.34 m	37.3 ± 0.58 c	82.0 ± 1.00 g–m
		TRESOR	3	17.8 ± 0.61 f–i	39.7 ± 1.11 b–d	100.0 ± 0.00 a	39.7 ± 1.11 b–e	23.7 ± 0.58 m–q	86.3 ± 0.58 e–g
		VETTORE	3	21.5 ± 0.62 b–e	39.9 ± 0.38 b–d	100.0 ± 0.00 a	39.9 ± 0.38 b–e	29.3 ± 1.53 f	92.0 ± 1.00 d
		VIRGILIO	3	18.3 ± 0.25 e–i	35.2 ± 1.00 c–f	95.0 ± 5.00 a	33.4 ± 2.13 d–h	28.3 ± 0.58 f–i	87.7 ± 1.15 d–f
		SUMAI #3	3	5.1 ± 0.06 p	2.6 ± 0.21 i	50.0 ± 5.00 c	1.30 ± 0.05 n	41.0 ± 0.00 a	127.0 ± 1.00 a
		GAMENYA	3	27.2 ± 0.36 a	55.1 ± 1.40 a	100.0 ± 0.00 a	55.1 ± 1.40 a	41.7 ± 0.58 a	106.7 ± 1.53 b
	RC	CRESO	3	13.7 ± 0.20 f–g	36.8 ± 1.17 bc	100.0 ± 0.00 a	36.8 ± 1.21 bc	28.7 ± 0.58 cd	75.0 ± 1.00 f–h
		DUILIO	3	20.4 ± 0.21 a–c	35.2 ± 0.25 b–e	100.0 ± 0.00 a	35.2 ± 0.25 bc	21.3 ± 0.58 hl	81.0 ± 1.00 de
		DUPRI”	3	15.1 ± 0.70 d–g	10.4 ± 1.06 f–g	66.7 ± 7.64 b	6.9 ± 1.06 f–g	25.7 ± 1.53 d–g	73.0 ± 2.00 h
		DYLAN	3	12.6 ± 0.36 f–g	13.5 ± 0.86 d–f	70.0 ± 5.00 b	9.5 ± 0.46 d–f	27.3 ± 0.58 c–e	81.3 ± 1.15 d
		FORTORE	3	18.5 ± 1.50 b–e	34.0 ± 5.80 b–e	100.0 ± 0.00 a	34.0 ± 2.19 bc	23.0 ± 1.00 f–i	88.3 ± 1.53 c
		GRECALE	3	20.6 ± 0.56 a–c	28.4 ± 1.07 c–e	96.7 ± 2.89 a	27.5 ± 0.20 c–e	23.0 ± 0.00 f–i	79.7 ± 1.53 d–f
		IRIDE	3	14.3 ± 1.90 e–g	38.2 ± 6.50 bc	98.3 ± 2.89 a	37.6 ± 6.50 bc	22.7 ± 0.58 f–i	75.3 ± 0.58 e–h
		ITALO	3	21.4 ± 1.10 ab	39.9 ± 1.66 bc	100.0 ± 0.00 a	39.9 ± 0.40 b	20.3 ± 0.58 il	81.3 ± 1.53 d
		MERIDIANO	3	16.6 ± 1.21 c–f	38.7 ± 1.11 bc	98.3 ± 3.54 a	38.0 ± 0.56 bc	29.0 ± 1.00 c	80.7 ± 2.52 d–f
		SAADI	3	22.1 ± 0.50 ab	41.5 ± 0.95 b	100.0 ± 0.00 a	41.5 ± 1.06 b	23.7 ± 1.53 f–i	73.3 ± 1.53 gh
		SARAGOLLA	3	13.9 ± 1.41 f–g	23.2 ± 4.56 e	91.7 ± 7.64 a	21.3 ± 6.13 e	23.7 ± 0.58 f–i	80.7 ± 2.52 d–f
		SIMETO	3	19.9 ± 0.75 bc	30.9 ± 5.84 b–e	90.0 ± 10.00 a	27.8 ± 2.30 c–e	22.3 ± 0.58 g–i	78.7 ± 1.53 d–g
		TIZIANA	3	11.7 ± 1.04 g	12.3 ± 0.71 fg	67.0 ± 7.55 b	8.2 ± 1.12 f	32.3 ± 1.53 b	80.0 ± 1.00 d–f
		TRESOR	3	16.4 ± 3.23 c–f	33.7 ± 6.50 b–e	98.3 ± 2.89 a	33.1 ± 6.85 bc	19.0 ± 1.00 l	78.3 ± 1.53 d–h
		VETTORE	3	19.3 ± 1.06 b–d	37.6 ± 1.12 bc	100.0 ± 0.00 a	37.6 ± 0.65 bc	26.0 ± 1.00 c–f	88.0 ± 2.00 c
		VIRGILIO	3	16.3 ± 0.36 c–f	23.8 ± 0.64 d–e	91.7 ± 7.64 a	21.9 ± 0.50 d–e	24.3 ± 0.58 e–h	87.7 ± 1.15 c
		SUMAI #3	3	4.5 ± 0.20 h	1.8 ± 0.10 g	45.0 ± 5.00 c	0.8 ± 0.07 g	36.3 ± 0.58 a	123.4 ± 0.79 a
		GAMENYA	3	24.6 ± 0.50 a	53.3 ± 1.45 a	100.0 ± 0.00 a	53.3 ± 1.06 a	37.3 ± 0.58 a	102.4 ± 0.20 b

^1^ Numerosity. Data are means of three replicates ±SD. Data in the same column followed by different letters are significantly different (Student–Newman–Keul test, *p* ≤ 0.001).

**Table 4:** Factorial ANOVA of the agronomic and qualitative traits of 16 Italian durum wheat cultivars and two reference cultivars of bread wheat evaluated for their susceptibility to Fusarium head blight (FHB). The test was performed in two different sites in northern and southern Italy, respectively: Eraclea (Veneto region) and Reggio Calabria (Calabria region).

Factors of Variability	Site	Cultivar	N^1^	Grain Yield (t ha^−1^)	Physiological Maturity (days from 01/04)	Hectoliter Weight (kg hL^−1^)	Thousand Kernels Weight (g)	Protein Content (% d.m.)
**Site (A)**				***	***	**	***	***
	Eraclea		54	4.6 ± 0.70	73.0 ± 3.19	79.3 ± 2.44	40.6 ± 5.66	12.9 ± 1.26
	RC		54	4.0 ± 0.67	63.1 ± 2.82	79.6 ± 2.84	39.5 ± 6.05	13.1 ± 1.57
**Cultivar (B)**				***	***	***	***	***
		CRESO	6	3.6 ± 0.28 gh	71.7 ± 5.96 b	81.3 ± 0.24 a–c	46.1 ± 0.27 c	13.5 ± 0.37 d
		DUILIO	6	4.0 ± 0.14 e–g	62.5 ± 4.68 l	81.4 ± 1.34 ab	46.7 ± 0.52 b	12.9 ± 0.64 g
		DUPRÌ	6	3.6 ± 0.98 gh	69.0 ± 4.6 c–f	78.3 ± 0.08 hi	36.9 ± 0.83 l	16.2 ± 0.18 a
		DYLAN	6	4.9 ± 0.57 bc	68.2 ± 4.96 d–g	81.3 ± 0.46 a–c	46.0 ± 0.30 c	11.9 ± 0.53 l
		FORTORE	6	3.6 ± 0.3 gh	66.7 ± 5.20 e–i	78.8 ± 0.13 g–i	37.2 ± 0.18 il	12.4 ± 0.14 f–h
		GRECALE	6	4.2 ± 0.4 ef	64.2 ± 5.42 il	80.3 ± 0.13 a–f	37.4 ± 0.32 i	12.7 ± 0.21 fg
		IRIDE	6	4.7 ± 0.42 cd	66.3 ± 5.96 f–i	81.1 ± 1.45 a–d	40.7 ± 0.46 g	12.4 ± 0.19 hi
		ITALO	6	3.8 ± 0.39 f–h	68.5 ± 5.32 d–g	80.1 ± 0.29 b–g	37.1 ± 5.12 il	12.2 ± 0.19 i
		MERIDIANO	6	4.8 ± 0.26 b–d	67.7 ± 4.80 d–h	78.1 ± 0.18 i	44.4 ± 0.42 d	12.7 ± 0.44 g
		SAADI	6	3.4 ± 0.47 h	70.0 ± 6.23 b–d	81.2 ± 2.02 a–c	32.7 ± 0.59 n	15.6 ± 0.99 b
		SARAGOLLA	6	4.1 ± 0.35 e–g	66.0 ± 5.55 g–i	80.8 ± 1.59 a–e	42.3 ± 0.19 f	12.6 ± 0.31 fg
		SIMETO	6	3.9 ± 0.33 e–g	67.3 ± 5.89 d–h	79.8 ± 1.57 d–g	47.9 ± 0.22 a	13.2 ± 0.16 e
		TIZIANA	6	5.2 ± 0.58 b	71.2 ± 5.38 bc	80.0 ± 0.66 c–g	46.1 ± 1.12 c	12.0 ± 0.15 l
		TRESOR	6	4.1 ± 0.55 e–g	65.2 ± 5.78 hi	81.7 ± 0.89 a	40.2 ± 3.41 h	15.4 ± 1.04 c
		VETTORE	6	4.4 ± 0.21 de	71.3 ± 5.16 bc	79.6 ± 0.91 e–g	36.8 ± 0.92 l	13.2 ± 0.16 e
		VIRGILIO	6	5.1 ± 0.48 bc	69.2 ± 5.71 b–e	79.4 ± 0.12 f–h	43.0 ± 0.27 e	12.6 ± 0.48 h
		SUMAI #3	6	5.6 ± 0.38 a	73.8 ± 6.49 a	74.7 ± 0.29 l	33.2 ± 0.10 m	11.0 ± 0.28 n
		GAMENYA	6	4.6 ± 0.21 cd	65.8 ± 6.11 g–i	71.7 ± 0.20 m	26.5 ± 0.10 o	11.7 ± 0.24 m
**A x B**				***	***	***	***	***
	Eraclea	CRESO	3	3.8 ± 0.18 m–p	77.0 ± 1.00 ab	81.1 ± 0.06 a–f	46.0 ± 0.15 c	13.9 ± 0.06 e
		DUILIO	3	3.9 ± 0.08 i–p	66.7 ± 1.53 h–l	80.2 ± 0.10 c–l	46.3 ± 0.06 c	13.4 ± 0.06 f
		DUPRÌ	3	4.5 ± 0.23 f–m	73.0 ± 2.00 c–e	78.3 ± 0.10 h–m	37.6 ± 0.15 m	16.1 ± 0.06 b
		DYLAN	3	5.4 ± 0.09 a–d	72.7 ± 0.58 c–f	80.9 ± 0.15 a–g	46.3 ± 0.15 c	11.4 ± 0.06 q
		FORTORE	3	3.5 ± 0.17 pq	71.3 ± 1.15 e–g	78.9 ± 0.15 g–m	37.3 ± 0.20 m	12.5 ± 0.1 l
		GRECALE	3	4.5 ± 0.16 f–l	69.0 ±1.00 f–h	80.4 ± 0.12 c–h	37.7 ± 0.2 m	12.5 ± 0.06 lm
		IRIDE	3	5.0 ± 0.15 c–g	71.7 ± 1.53 e–g	79.9 ± 0.10 d–m	40.3 ± 0.2 l	12.2 ± 0.1 l–o
		ITALO	3	4.1 ± 0.14 h–p	73.3 ± 0.58 b–e	80.3 ± 0.20 c–i	41.7 ± 0.35 hi	12.0 ± 0.06 n–p
		MERIDIANO	3	5.0 ± 0.12 c–f	72.0 ± 1.00 d–f	78.1 ± 0.12 lm	44.1 ± 0.15 e	13.1 ± 0.10 f–i
		SAADI	3	3.8 ± 0.18 l–p	75.7 ± 0.58 b–d	82.4 ± 0.12 ab	33.2 ± 0.15 o	14.7 ± 0.10 c
		SARAGOLLA	3	4.4 ± 0.19 f–n	71.0 ± 1.00 e–g	79.3 ± 0.12 f–m	42.1 ± 0.15 gh	12.9 ± 0.10 hi
		SIMETO	3	4.2 ± 0.17 h–p	72.7 ± 0.58 c–f	78.4 ± 0.12 h–m	48.1 ± 0.10 a	13.0 ± 0.06 g–i
		TIZIANA	3	5.7 ± 0.15 ab	76.0 ± 1.00 bc	80.6 ± 0.10 b–g	47.1 ± 0.12 b	11.8 ± 0.06 p
		TRESOR	3	4.6 ± 0.13 f–l	70.3 ± 1.53 e–g	81.5 ± 0.10 a–e	43.3 ± 0.10 f	14.4 ± 0.00 d
		VETTORE	3	4.5 ± 0.14 f–m	76.0 ± 1.00 bc	80.2 ± 0.12 c–l	37.4 ± 0.12 m	13.1 ± 0.06 f–i
		VIRGILIO	3	5.5 ± 0.16 a–c	74.3 ± 0.58 b–e	79.5 ± 0.10 e–m	43.2 ± 0.06 f	12.2 ± 0.1 l–o
		SUMAI #3	3	6.0 ± 0.09 a	79.7 ± 1.53 a	74.9 ± 0.10 n	33.2 ± 0.06 o	10.8 ± 0.06 r
		GAMENYA	3	4.5 ± 0.21 f–l	71.3 ± 1.53 e–g	71.8 ± 0.15 o	26.5 ± 0.06 q	11.9 ± 0.06 n–p
	RC	CRESO	3	3.5 ± 0.30 ef	66.3 ± 1.53 ab	81.5 ± 0.15 a–d	46.2 ± 0.35 b	13.2 ± 0.10 bc
		DUILIO	3	4.2 ± 0.10 be	58.3 ± 0.58 h	82.6 ± 0.12 a	47.2 ± 0.10 a	12.3 ± 0.06 e
		DUPRÌ	3	2.7 ± 0.18 g	65.0 ± 1.00 a–c	78.2 ± 0.00 e	36.1 ± 0.15 g–i	16.3 ± 0.15 a
		DYLAN	3	4.4 ± 0.27 bd	63.7 ± 0.58 b–e	81.7 ± 0.15 a–d	45.8 ± 0.20 bc	12.4 ± 0.10 e
		FORTORE	3	3.8 ± 0.31 cf	62.0 ± 1.00 cg	78.8 ± 0.12 de	37.10 ± 0.10 gh	12.4 ± 0.15 e
		GRECALE	3	3.9 ± 0.13 cf	59.3 ± 1.53 gh	80.2 ± 0.12 a–e	37.2 ± 0.06 h	12.8 ± 0.06 cd
		IRIDE	3	4.4 ± 0.36 bd	61.0 ± 1.00 dh	82.2 ± 1.13 ab	41.1 ± 0.10 f	12.5 ± 0.10 de
		ITALO	3	3.5 ± 0.19 eg	63.7 ± 0.58 b–f	79.9 ± 0.15 a–e	32.4 ± 0.10 lm	12.4 ± 0.06 e
		MERIDIANO	3	4.6 ± 0.20 ac	63.3 ± 0.58 b–f	78.0 ± 0.21 e	44.8 ± 0.10 d	12.3 ± 0.00 e
		SAADI	3	3.0 ± 0.21 fg	64.3 ± 0.58 b–d	80.0 ± 2.37 a–e	32.2 ± 0.32 m	16.5 ± 0.10 a
		SARAGOLLA	3	3.8 ± 0.20 cf	61.0 ± 1.00 d–h	82.2 ± 0.15 ab	42.4 ± 0.10 e	12.4 ± 0.15 e
		SIMETO	3	3.7 ± 0.28 d–f	62.0 ± 1.00 c–g	81.2 ± 0.15 a–d	47.7 ± 0.12 a	13.3 ± 0.10 b
		TIZIANA	3	4.7 ± 0.15 ab	66.3 ± 1.15 ab	79.4 ± 0.10 b–e	45.1 ± 0.10 cd	12.1 ± 0.00 e
		TRESOR	3	3.6 ± 0.17 d–f	60.0 ± 1.00 f–h	81.8 ± 1.39 a–c	37.1 ± 0.40 h	16.3 ± 0.10 a
		VETTORE	3	4.3 ± 0.21 b–e	66.7 ± 0.58 ab	79.0 ± 0.98 c–e	36.1 ± 0.92 i	13.3 ± 0.20 b
		VIRGILIO	3	4.7 ± 0.19 ac	64.0 ± 1.00 b–e	79.3 ± 0.06 b–e	42.8 ± 0.20 e	13.1 ± 0.12 bc
		SUMAI #3	3	5.3 ± 0.17 a	68.0 ± 1.00 a	74.5 ± 0.31 f	33.2 ± 0.15 l	11.2 ± 0.15 f
		GAMENYA	3	4.7 ± 0.20ab	60.3 ± 0.58 e–h	71.5 ± 0.12 g	26.4 ± 0.10 n	11.5 ± 0.20 f

N^1^ Numerosity. Data are means of three replicates ±SD. Data in the same column followed by different letters are significantly different (Student–Newman–Keul test, *p* ≤ 0.001).

## Data Availability

The data presented in this study are available on request from the corresponding author.
